# How to measure the effectiveness of healthcare providers acting as an ‘anchor institution’: a case study of the NHS in Greater Manchester, England

**DOI:** 10.1136/bmjopen-2025-111328

**Published:** 2026-03-18

**Authors:** Vasudha Wattal, Christine Camacho, Anna Gkiouleka, John Alexander Ford, Luke Aaron Munford

**Affiliations:** 1 Health Organisation, Policy, and Economics (HOPE) Research Group, Division of Population Health, Health Services Research and Primary Care, The University of Manchester, Manchester, UK; 2 Wolfson Institute of Population Health, Queen Mary University of London, London, UK; 3 Department of Social Work and Social Policy, University of Strathclyde, Glasgow, UK

**Keywords:** PUBLIC HEALTH, Health policy, Health Equity

## Abstract

**Abstract:**

**Objectives:**

To improve social determinants of health, healthcare organisations can support societal and economic goals in their role as anchor institutions (large organisations with an important presence and ties to a place). In England, the National Health Service (NHS) Long Term Plan highlighted the role of the NHS as an Anchor. Despite a clear policy mandate on this, less is known about specific indicators to measure and benchmark anchor performance. A set of metrics was developed to quantify anchor activity using the Greater Manchester (GM) region in England as a case study.

**Design:**

Descriptive cross-sectional study.

**Setting:**

Data were received on employment and procurement for the financial year 2022/2023 from NHS trusts located in GM.

**Primary and secondary outcome measures:**

Performance against two anchor metrics, local spending and employment, was assessed. ‘Local’ was defined as the Integrated Care Board (ICB) footprint in which the trusts are located. The proportion of procurement spend to the local economy was derived from procurement data. Employment data was aggregated by ethnicity codes and deprivation levels and compared with ICB-level ethnicity and deprivation profiles using the Index of Multiple Deprivation based on 2021 Census data.

**Results:**

The included trusts employed 65 597 residents of GM and spent £389 million on local procurement, demonstrating their importance as anchor organisations. Considerable variation was observed between trusts in local spending, ranging from 6.4% (95% CI 6.4% to 6.41%) to 52.7% (95% CI 52.69% to 52.72%) (with the mean at 21%). The percentage of locally employed staff ranged from 82.7% (95% CI 81.45% to 83.90%) to 89.5% (95% CI 89.12% to 89.95%). All trusts demonstrate strong workforce representation from minoritised ethnic groups, but most employed a lower proportion of staff from the most deprived areas than expected based on the local population profile.

**Conclusions:**

It is feasible to quantify aspects of anchor activity using routine NHS data, and meaningful variation exists across trusts, even within a single health system. GM provides a useful case study, but further work is needed to embed anchor metrics in routine reporting and to extend measurement to other domains such as estates and sustainability.

**STRENGTHS AND LIMITATIONS OF THIS STUDY**

- A key strength of this study lies in its use of routinely available data to operationalise and test indicators of anchor activity in local healthcare organisations.
- By using consistent definitions, variation across organisations could be meaningfully interpreted.
- Limitations with respect to data availability across all local organisations and/or all anchor metrics mean that the full extent of potential anchor contributions could not be examined.

## Background

 Large organisations with an important presence and ties to a place can be referred to as ‘anchor institutions’.^1^ They are called as such since these organisations are unlikely to relocate and are effectively ‘anchored’ in their local community.^2^ Common examples include universities, local healthcare organisations such as National Health Service (NHS) trusts and local authorities; private sector organisations such as airports and large local businesses can also act as anchor institutions. A key characteristic of anchor institutions is their size, and while there is no quantification of this, the crucial factor is their influence over local economic development.^3^ Through purchasing goods and services from their local community, employment procedures and their use of their land and estates, anchor institutions can contribute to improving local wealth and health.^2 3^

In England, there have been growing concerns about persistent inequalities in income and health outcomes.^4^ People living in poverty are more likely to have poor health and recent trends in mortality have shown widening gaps linked with socioeconomic inequalities.^5 6^ Increasing the flow of wealth within a local area by harnessing the potential of anchor institutions is known as community wealth building.^2 7^ Localised place-based strategies to enhance economic conditions have the potential to improve health and well-being. A community wealth building programme in Preston, a relatively deprived city in the North West of England, led to improvement in life satisfaction, employment and economic measures which translated into improvements in mental health and well-being.^8 9^ Social value through public procurement can be attained by embedding relevant criteria for supplier selection and setting procurement specific goals, and social value goals according to place-based needs.^10^

The NHS is facing mounting pressures from rising demand and constrained resources, which has prompted an increased focus on disease prevention and improved well-being of local communities. The NHS 10 Year Health Plan emphasises that hospitals are expected to do more to support societal and economic goals in their role as anchor institutions.^11^ The NHS is the largest employer in the UK and one of the largest globally, employing more than 1.6 million people in the UK, and spending around £27 billion each year on goods and services in England.^2^ As such, it is well positioned to act as an anchor, through its procurement, employment, sustainability practices and estates.^12^ The NHS has committed to having a net zero carbon footprint by 2045^13^ and has introduced a minimum 10% social value into all its procurement contracts since April 2022.^14^ In addition to national policy initiatives, some local NHS organisations are taking actions to expand employment and skill development opportunities to people living in the most deprived areas.^15^ An example is the Connecting Communities with Health and Care Careers Programme in Leeds, which provides training opportunities to boost employability within communities facing disadvantage.^11^

Frameworks have been proposed that conceptualise anchor activity being organised across five domains, namely workforce, procurement, land and buildings, environmental sustainability and partnerships.^2 16^ However, less is known about what anchor actions look like in practice in terms of outcomes, and how and where should NHS organisations prioritise their actions.^2^ Without clear metrics and indicators of anchor-related performance, it is difficult for organisations to track progress, benchmark performance or integrate anchor activity into routine governance and accountability structures. In this study, we aim to bridge this gap through measurement of anchor activity of local NHS organisations in two domains—procurement and employment—and to describe what anchor activity might look like.

In this paper, Greater Manchester (GM) was used as a case study site to describe and quantify anchor activity in the NHS. GM is a metropolitan city region located in the North-West of England and has a population of 2.9 million. The GM Combined Authority (GMCA) is made up of ten local authorities. On average, GM has higher levels of deprivation, with 41% of working-age population living in the most deprived areas, compared with 20% nationally. The GMCA has co-terminus boundaries with the GM Integrated Care Board (ICB) which is the NHS organisation responsible for commissioning and co-ordinating health services to meet the needs of the local population.^17^

## Data and methods

The role of NHS trusts as anchor institutions was examined using two anchor domains: local spending and local employment. Following consultation with stakeholders, ‘local’ was defined as the geographic footprint of the ICB in which each trust is located (in this case, GM). A total of nine NHS trusts operates within the GM region, including two specialist trusts. Employment and procurement data were requested and received from individual trusts. Although data on estates and sustainability were also sought, no trusts were able to provide recent or complete data.

For local procurement, non-pay spend data for the 2022/2023 financial year were provided by all nine trusts. Supplier postcodes were mapped to Lower Super Output Areas (LSOAs) and subsequently aggregated to ICB level. An indicator was derived for each trust representing the proportion of non-pay spend within the GM ICB footprint. Additional indicators were calculated for the North West region (including GM ICB, Cheshire and Merseyside ICB, and Lancashire and South Cumbria ICB) and for the rest of England. Records for suppliers and staff located outside England were excluded from the analysis. To the best of our knowledge, the procurement data were complete.

Employment data were received from seven trusts anonymised at individual employee level, including area of residence (LSOA/Middle Super Output Area (MSOA)) and ethnicity. Ethnicity codes were grouped into five categories (Asian, Black, Mixed, Other and White) in line with the Office for National Statistics census classification.^18^ Trust-level workforce profiles were compared with the GM ICB working-age population (aged 16–64), as reported in the 2021 Census.^19^

Deprivation was assessed using the Index of Multiple Deprivation (IMD), a nationally standardised measure of relative deprivation at neighbourhood level in England.^20^ IMD deciles were matched to each MSOA or LSOA of residence, reclassified into quintiles (with Quintile 1 representing the most deprived areas), and aggregated to determine the distribution of trust staff across deprivation quintiles. These were then compared with the deprivation profile of the GM ICB working age (16–64) population from the 2021 census.

## Results

### Procurement spending

**Figure 1:**
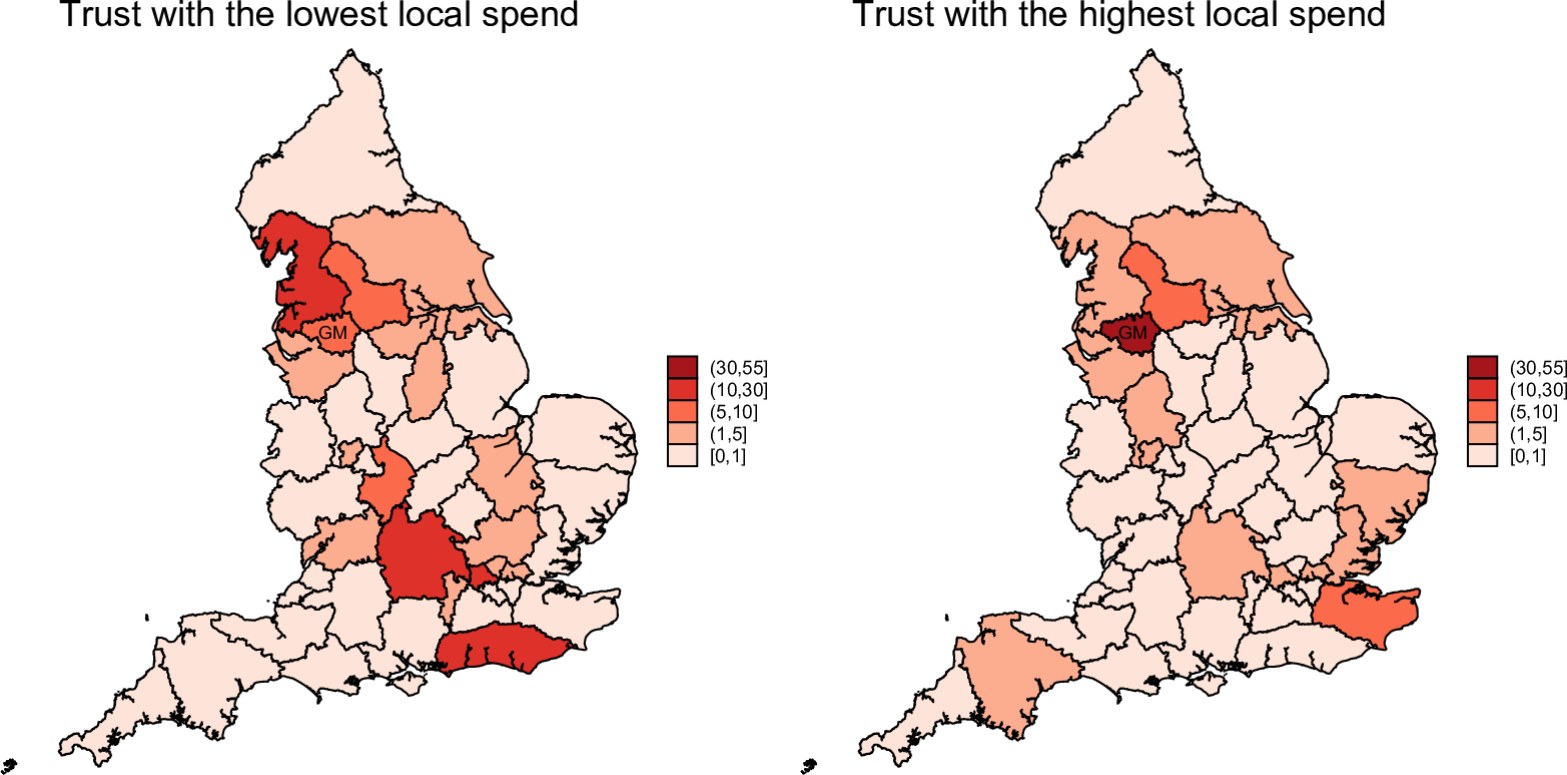
Trust level spending distribution across ICBs in England. ICBs, Integrated Care Boards.

Across the nine NHS trusts in GM, total non-pay spend in 2022/2023 amounted to £1.83 billion, of which £389 million (21%) was spent within the GM ICB footprint. On average, local spend per trust was £4.32 million (SD: £6.16 million), although considerable variation was observed, ranging from 6.4% to 52.7% of total non-pay expenditure (table 1). Spatial procurement patterns for the trusts with the lowest and highest proportions of local spend are illustrated in figure 1. This spatial pattern supports visualisation of these variations, as the trust with the lowest local spend (left map) procures amounting to 10%–30% of its’ spend from its immediate neighbour (Lancashire and South Cumbria ICB) and from the South East. Whereas, for the trust with the highest local spend (right map), its’ procurement from ICBs outside of GM ICB remains under 10%. When procurement within the broader North West region is considered, variation narrows, with trust spending in the region ranging from 20.2% to 58.4%.

**Table 1:** Overview of procurement spending by the area in which the money was spent

	GM ICB spend % (95% CI)	North West region (%) (95% CI)	Rest of England (%) (95% CI)
Trust A	52.71 (52.69 to 52.72)	58.44 (58.43 to 58.45)	41.56 (41.55 to 41.57)
Trust B	41.67 (41.67 to 41.68)	46.25 (46.25 to 46.26)	53.75 (53.74 to 53.75)
Trust C	19.11 (19.1 to 19.12)	40.5 (40.49 to 40.51)	59.5 (59.49 to 59.51)
Trust D	17.6 (17.62 to 17.62)	32.44 (32.43 to 32.44)	67.56 (67.56 to 67.57)
Trust E	16.85 (16.84 to 16.85)	28.72 (28.71 to 28.73)	71.28 (71.27 to 71.29)
Trust F	15.43 (15.42 to 15.44)	36.39 (36.37 to 36.4)	63.61 (63.6 to 63.63)
Trust G	11.5 (11.49 to 11.51)	25.18 (25.16 to 25.19)	74.82 (74.81 to 74.84)
Trust H	7.76 (7.75 to 7.76)	56.07 (56.06 to 56.08)	43.93 (43.92 to 43.94)
Trust I	6.4 (6.4 to 6.41)	20.2 (20.19 to 20.21)	79.8 (79.79 to 79.81)
GM Trust mean	21	38.24	61.76

GM, Greater Manchester; ICB, Integrated Care Board.

### Workforce

Across the seven trusts that provided employment data, a total of 75 405 staff were employed, of whom 65 597 (87%) were residents of the GM ICB footprint. The proportion of locally employed staff ranged from 82.7% (95% CI 81.45% to 83.90%) to 89.5% (95% CI 89.12% to 89.95%) (table 2).

**Table 2:** Trust-wise share of overall workforce resident in GM

	Share of local staff employed (95% CI)
Trust A	85.1% (84.02% to 86.26%)
Trust B	89.5% (89.12% to 89.95%)
Trust C	83.9% (83.11% to 84.76%)
Trust D	86.8% (86.43% to 87.23%)
Trust E	85.7% (84.18% to 85.93%)
Trust G	88.9% (88.05% to 89.78%)
Trust I	82.7% (81.45% to 83.90%)
GM Trust mean	86.0%

GM, Greater Manchester.

The proportion of staff residing in the most deprived areas of GM (deprivation quintile 1) ranged from 22.6% to 42.4% across trusts, with most employing a lower share of staff from these areas than would be expected based on the population distribution (41.4% of GM’s working-age population lives in quintile 1). The full distribution across deprivation quintiles is shown in table 3.

**Table 3:** Trust-wise employment by deprivation quintile compared with the GM ICB population, in percentages of overall workforce resident in GM

	1-most deprived	2	3	4	5-least deprived
Trust A	30.57	22.89	17.46	17.85	11.24
Trust B	42.38	25.25	12.18	11.07	9.12
Trust C	39.31	25.86	12.47	11.37	10.99
Trust D	33.93	23.73	13.43	14.87	14.04
Trust E	22.59	21.68	20.12	15.46	20.15
Trust G	41.84	24.32	22.20	4.35	7.29
Trust I	25.87	20.96	18.58	18.77	15.82
GM ICB population	41.36	22.0	13.45	12.86	10.34

GM, Greater Manchester; ICB, Integrated Care Board.

Compared with the GM working-age population, most trusts reported higher proportions of local staff from Asian, Black, Mixed and Other ethnic backgrounds (table 4). This indicates relatively strong representation of minoritised ethnic groups. However, variation across trusts was observed, and in some cases, proportions of White staff remained substantially higher than the GM average. The share of staff for whom ethnicity data were missing ranged from 1.3% to 9.1%.

**Table 4:** Trust-wise employment by ethnicity compared with the GM ICB population, in percentages of overall workforce resident in GM

	Asian	Black	Mixed	Other	White	Unknown
Trust A	7.22	7.79	2.50	0.86	81.64	1.3
Trust B	15.01	6.03	1.86	2.38	74.73	1.7
Trust C	7.34	15.02	2.65	1.43	73.57	5.2
Trust D	17.25	8.71	2.57	2.71	68.76	9.1
Trust E	16.36	5.48	1.83	2.97	73.35	2.4
Trust G	17.34	6.40	1.99	2.03	72.24	1.6
Trust I	9.77	5.06	2.50	2.44	80.23	3.8
GM ICB population	13.94	4.52	1.93	2.29	77.32	–

GM, Greater Manchester; ICB, Integrated Care Board.

While there is no single benchmark for what constitutes a high-performing anchor institution, the combined metrics of local procurement, employment of local residents, workforce diversity and employment from deprived areas allow for a comparative view across trusts. Trust B emerges as a relatively strong performer across multiple dimensions (table 5). It has the second-highest proportion of local procurement spending (41.7%) and the highest local employment rate (89.5%) among all trusts. It also demonstrates the highest percentage of staff from the most deprived areas (42.4%), closely aligning with the GM population average (41.4%). Additionally, 25.3% of its workforce is from minoritised ethnic groups, above the GM population average.

**Table 5:** Trust-wise performance on anchor-related metrics, a summary

Trust	Local spend (%)	Local employment (%)	Staff living in most deprived IMD quintile (%)	Staff from minoritised ethnic groups (%)
Trust A	52.7	85.1	30.6	18.4
Trust B	41.7	89.5	42.4	25.3
Trust C	19.1	83.9	39.3	26.4
Trust D	17.6	86.8	33.9	31.2
Trust E	16.9	85.1	22.6	26.6
Trust G	11.5	88.9	41.8	27.8
Trust I	6.4	82.7	25.9	19.8

IMD, Index of Multiple Deprivation.

Other trusts show more mixed profiles. Trust A has the highest level of local procurement, but more limited diversity and deprivation representation. Trusts C, D and G perform well on workforce diversity and employment from deprived areas, though their local procurement levels are notably lower. Trust I, by contrast, ranks consistently lower across all four dimensions. These patterns suggest that anchor performance varies substantially across trusts, and that strengths in one domain (eg, local employment) do not necessarily align with strengths in another (eg, local procurement).

## Discussion

### Main findings

This is the first study to operationalise metrics to measure anchor activity in NHS organisations. It demonstrates the feasibility of measuring anchor activity in NHS organisations using routine employment and procurement data. Using GM as a case study, metrics were developed and applied to quantify two domains of anchor activity: local employment and local spending. Substantial variation was observed between trusts, with local procurement ranging from 6.4% to 52.7% of total non-pay spend, and the proportion of staff residing within the local ICB area ranging from 82.7% to 89.5%. The observed variation in local spend across trusts may be on account of inclusion of two specialist trusts which may be required to buy specialised high value equipment through procurement frameworks such as the NHS Supply Chain,^21^ and as such have less autonomy. Across all participating trusts, minoritised ethnic groups appeared well-represented relative to the local population, although this may reflect patterns of international recruitment and occupational clustering. Data on estates and environmental sustainability, two other key dimensions of the anchor role, were not available, highlighting ongoing limitations in data infrastructure and accountability in these areas.

### Strengths and weaknesses

A key strength of this study lies in its use of routinely available data to operationalise and test indicators of anchor activity in NHS trusts. Employment and procurement data were obtained directly from trusts and analysed against population-level deprivation and ethnicity profiles, enabling a comparative assessment of local performance. The application of consistent definitions across all participating organisations also allowed variation to be meaningfully interpreted within the same health system context. In addition, the use of GM, an area with an established history of devolution and anchor collaboration, provides a relevant and policy-relevant setting for testing the feasibility of measurement approaches.

However, several limitations must be acknowledged. Complete employment data were available from only seven of the nine GM trusts, and missing information on staff ethnicity within submitted datasets limited the ability to draw firm conclusions on representativeness. While procurement data were received from all trusts, the analysis was restricted to spend location and did not include data on supplier type, ownership or social value content. Data was requested on two other key domains of anchor activity, use of estates (measured in terms of number of bookings by community/voluntary sector) and environmental sustainability (measured as estimated annual carbon dioxide emission for the Trust). However, none of the trusts were able to report data on estates, and all trusts reported data on the sustainability indicator which was part of net zero ‘baseline’ and hence could not be compared with the remaining analysis which was more recent. As such, the analysis does not capture the full breadth of potential anchor contributions. GM is highly urbanised and densely populated, while being well-connected through strong transport infrastructure, factors contributing to supply chain efficiency and workforce mobility. While this study does not intend to report generalisable findings for all of England, consideration of the local context is important, and less urban ICBs may generate different findings from these anchor measures.

As this was a retrospective study, we were limited by data we thought might be routinely collected in the NHS related to the five anchor pillars. If this exercise was to be done prospectively, we would want to collect nuanced data on workforce parameters such as educational level, gender, access to flexible working, promotion rates, access to apprenticeships, internships and entry-level roles, etc. A broader range of demographics and socioeconomic characteristics would allow examination of workforce representation, access to employment and training opportunities, and career progression and workplace support.

### Strengths/weaknesses in relation to other studies

Most existing literature on anchor institutions in the NHS has focused on conceptual frameworks, case studies, or policy guidance, with limited application of quantitative metrics to assess organisational performance. While some local evaluations have explored aspects of anchor activity, such as procurement spend or workforce inclusion, these analyses have typically been fragmented, methodologically diverse or unpublished.^22^ Toolkits such as the UCL Partners measurement framework provide a structured menu of potential indicators, but these have not been applied systematically or benchmarked across organisations.^16^ Recent studies have emphasised the need to move beyond descriptive accounts and develop consistent, data-driven approaches to monitor anchor outcomes.^2 12 23^

A notable example of prior quantitative analysis is the Leeds City Region case study, which examined anchor activity across ten major public-sector institutions, including NHS trusts, local authorities and universities.^24^ That study estimated a combined annual procurement spend (non-pay) of £1.4 billion, of which approximately 50% was retained within the local region. While the institutions analysed extended beyond the NHS, the focus on procurement spend makes the findings directly comparable. In contrast, the present study examined non-pay procurement within NHS trusts only, identifying a lower mean share of local spend at 21%. This difference may reflect structural and contextual factors such as procurement autonomy, variations across public sector organisations, or local supply chain availability. Nevertheless, both studies illustrate the feasibility of quantifying anchor activity at system level and underscore the need for standardised metrics to support benchmarking and improvement across organisations and regions.

### Meaning of study and policy implications

The variation observed across NHS trusts in both local procurement and local employment suggests that anchor activity is not being pursued consistently, even within a single integrated care system. These differences may reflect variation in local recruitment strategies, procurement autonomy, organisational leadership or governance arrangements. It is also possible that some trusts are engaging in anchor-like activities without a formal strategy, while others may have made high-level commitments without embedding delivery mechanisms. The apparent over-representation of minoritised ethnic groups in some trust workforces may be attributable to international recruitment, particularly in nursing,^25^ or the concentration of local ethnic minority communities in lower-paid roles such as estates and facilities. These patterns highlight the need to distinguish between passive indicators of anchor activity and deliberate strategies aimed at inclusive economic development.

A core principle of community wealth building is the intentional redirection of economic flows into local communities. While all participating trusts employed a high proportion of local residents, an anchor approach grounded in community wealth building would also involve targeted recruitment and skills pathways for individuals who are unemployed or economically inactive. Although some initiatives of this kind are in place within GM,^22^ the available data do not allow differentiation between local recruitment, internal migration from other parts of the UK and international hiring. In particular, the impact of large-scale international recruitment may confound interpretations of workforce diversity, potentially masking structural inequalities within the labour market.

In the case of procurement, low local spend does not necessarily imply a lack of intent. The Public Services (Social Value) Act of 2012 (enforced in 2013) requires public authorities to consider the creation of economic, social and environmental value through their procurement strategies, and the more recent Procurement Act of 2023 (enforced in 2025) strengthens these obligations.^26 27^ However, structural constraints, including the central procurement of specialist equipment, national frameworks and the commissioning of specialist trusts, may limit the autonomy of some organisations in shaping their supply chains.^28^ Some procurement also involves a national competitive bidding process, and in these instances, local may not necessarily be the cheapest.^12^ There may also be limited availability of local suppliers and/or their capacity to participate in bidding. However, without greater transparency and governance, it is difficult to determine whether low local spend reflects external constraints or the absence of anchor priorities within procurement strategy. An additional dimension to consider may be the distribution of local spend across providers of different capacities and whether this is able to boost engagement from small and medium enterprises.

The findings of this study have several implications for policy and practice. First, the absence of routine and comparable data across anchor domains limits both local accountability and the ability to benchmark performance across organisations. Improving data quality, particularly for staff ethnicity, will be essential if anchor metrics are to be integrated into routine reporting. Second, while tools such as the NHS Anchor Metrics Toolkit provide a useful starting point, they currently lack benchmarks or performance thresholds, leaving organisations to interpret success independently. Third, integrated care systems may have an important role in coordinating anchor action across trusts and supporting shared learning. However, the ability of ICSs to fulfil this role will depend on sustained capacity and alignment with other strategic priorities, particularly in light of ongoing organisational restructuring.

Strengthening the role of NHS trusts as anchor organisations requires leadership, governance systems and accountability mechanisms. The Health Anchors Learning Network, initially set up by the Health Foundation and NHS England in 2021, has been collectively exploring the anchor role across 1000 member organisations through sharing resources and best practices.^29^ Senior-level commitment remains a critical enabler of anchor action, particularly in identifying actionable opportunities and developing locally appropriate metrics.^12 23^ Although one of the stated aims of integrated care systems is to support local social and economic development, further evaluation is needed to understand the extent to which this ambition is being realised through partnerships with local anchor institutions.^30^

In addition to leadership and coordination, deliberate attention to equity is essential if the anchor role is to deliver meaningful social value. Allen *et al* propose five equity principles to guide hospitals in maximising their impact as anchor institutions, highlighting that social value is not automatically generated through procurement or employment alone, but must be intentionally designed into institutional practice.^31^ These principles emphasise the importance of focusing on those with the greatest need, using data to identify and address inequities, and embedding equity into governance and accountability structures. The variation observed in this study—particularly in workforce diversity and local procurement—suggests that without such intentionality, anchor activities may be inconsistently applied or risk reinforcing existing disparities. Aligning anchor strategies with equity-focused principles may help shift organisations from passive contributors to active agents of inclusive local development.

### Future research

This study raises several questions that merit further exploration. First, future research could assess how routinely collected NHS data might be leveraged to support the measurement of anchor activity across trusts. The metrics developed in this study, focusing on local procurement and local employment, could be applied systematically across different geographical contexts, including rural or mixed economies, to explore how local context shapes anchor performance.

Second, there is a need for a national benchmarking study that applies a consistent set of anchor indicators to all NHS trusts in England. Such work would help identify variation by trust type and size and support the development of performance thresholds or typologies to inform improvement efforts.

Third, further analysis is required to understand who benefits from anchor employment strategies. In particular, it remains unclear to what extent NHS recruitment supports local populations who are unemployed or economically inactive, versus attracting inward migration from other parts of the UK or internationally. While representation of minoritised ethnic groups appears high in the current analysis, further work is needed to assess the distribution of staff across pay bands and job roles in order to evaluate equity and progression within the workforce. We started with deprivation and ethnicity as starting points for dimensions along which to examine anchor employment strategies, as they are in line with broader NHS policy as set out in the 10 year plan.^11^ However, data is being captured on other staff characteristics such as country of birth, sexual orientation, religious belief, etc which is an area to explore in future research. While we did not have access to information on the supplier capacity, future analysis using a richer dataset could look into whether local procurement strategies are able to empower smaller businesses to participate in bids, etc.

Previous longitudinal research on anchor institutions (such as for-profit hospitals in the USA) has demonstrated that instability (due to closures) and small scale of operations may signal lower contributions to surrounding community through employment and procurements.^32^ Thus, measuring and monitoring of change in anchor activity over time is vital to examining changes in local economic and health outcomes. A larger-scale study encompassing multiple domains of NHS anchor activity, including estates and environmental sustainability, would be needed to provide a comprehensive assessment and support the development of a national reporting framework.

## Data Availability

Data may be obtained from a third party and are not publicly available.
