# Supplementary material for: How to measure the effectiveness of healthcare providers acting as an ‘anchor institution’: a case study of the NHS in Greater Manchester, England
Source: BMJ Open. 2026 Mar 18;16(3):e111328. doi: 10.1136/bmjopen-2025-111328 (PMC13007124; doi:10.1136/bmjopen-2025-111328)
Supplement: Supplementary file 1 [file bmjopen-2025-111328.reviewer_comments.pdf]

PEER REVIEW HISTORY

BMJ Open publishes all reviews undertaken for accepted manuscripts. Reviewers are asked to complete a checklist review form and are provided with free text boxes to elaborate on their assessment. These free text comments are reproduced below.

ARTICLE DETAILS

Title (Provisional)

How to measure the effectiveness of healthcare providers acting as an 'anchor institution': A case study of the NHS in Greater Manchester, England

Authors

Wattal, Vasudha; Camacho, Christine; Gkiouleka, Anna; Ford, John Alexander; Munford, Luke Aaron

---

VERSION 1 - REVIEW

**Reviewer**	**1**
**Name**	**Hughes, Ben**
**Affiliation**	**University of Bolton, School of Health and Society**
**Date**	**24-Oct-2025**
**COI**	**None**

Thank you for your manuscript and the opportunity to read such a strong paper on an important topic. I found that this paper is really interesting and while I wouldn't describe myself as an expert in qualitative research, you statistics provided were relevant and clear. The writing style used throughout the manuscript were appropriate and aided understanding, taking the reader through your discussion points step by step. There are key learning points, which are really important to highlight in order to understand impact and implications, are clearly staged. As a whole, the paper reads very well and I hope this will be a good foundation for further research in this area. As such, I have no suggestions for corrections/amendments and I recommend publication of the manuscript in its current form.

**Reviewer**	**2**
**Name**	**Herlitz, Lauren**
**Affiliation**	**UCL GOS Institute of Child Health, Population Policy and Practice**
**Date**	**04-Dec-2025**

COI None

This paper is well written. The method is clear but a bit thin. It would be good to have a little more on the justification of the data sought from trusts.

From reading the citations, it's not just ties to a place but its also the stability of their presence in a particular area - please can this be made clearer. Are there any other specific features that makes something an anchor institution? E.g. does it have to be over a certain number of employees?

Was data on procurement complete?

Were ethnicity categories self-reported? Is it possible to get migrant status? (Or was this not considered?). Or any information on gender or disabilities? Why only ethnicity?

Was it possible to get any information on the grades of staff employed?

Why were there such differences between trusts on local spending? Any possible explanations?

I'm not quite sure what story Figure 1 is telling?

Interested in whether there is anything geographically that might explain why Trust B is the strongest performer.

No trusts were able to provide recent or complete data on estates and sustainability. It would be good to know what information was sought as other researchers might like to try to replicate the methods. Was there any information about why this wasn't attainable that you could include in the discussion? 'Data infrastructure and accountability' are quite superficial explanations. Have any other studies been conducted which describe large institutions estates and environmental sustainability? For example, UCL, an anchor institution in central London, uses KPIs for sustainability. https://www.ucl.ac.uk/sustainable/about-us/commitments-and-strategy/ucl-sustainability-plan-2025-35

There's a lack of citations in the para starting "In the case of procurement…"

I would like to get a sense in the discussion of what would be the key indicators you would like if the data was available.

VERSION 1 - AUTHOR RESPONSE

Reviewer Comment	Authors Response
From reading the citations, it's not just ties to a place but its also the stability of their presence in a particular area - please can this be made clearer. Are there any other specific features that makes something an anchor	We have included the following text in the Background section to clarify the definition of anchors. [p.2]<br><br>"They are called as such since these organisations are unlikely to relocate and are

institution? E.g. does it have to be over a certain number of employees?	effectively 'anchored' in their local community (2). ... A key characteristic of anchor institutions is their size, and while there is no quantification of this, the crucial factor is their influence over local economic development (3)."
Was data on procurement complete?	We have added a sentence to reflect this in the methods section [p.3].<br>"To the best of our knowledge, the procurement data was complete."
Were ethnicity categories self-reported? Is it possible to get migrant status? (Or was this not considered?). Or any information on gender or disabilities? Why only ethnicity?	Yes, ethnicity data comes from Electronic Staff Record (ESR) and is self-reported. However, as mentioned in the methods section, the data has been grouped into five categories based on ONS census-based classification.<br><br>Additionally, in the discussion section "future research", we have now included the following text to explain our strategy and which other characteristics may be explored in future research. [p.11]<br><br>"We started with deprivation and ethnicity as starting points for dimensions along which to examine anchor employment strategies, as they are line with broader NHS policy as set out in the 10 year plan (11). However, data is being captured on other staff characteristics such as country of birth, sexual orientation, religious belief, etc. which is an area to explore in future research."
Was it possible to get any information on the grades of staff employed?	We did ask for pay grade information. However, as the data received was not complete or comparable across all trusts, it was omitted from the current analysis. We reached out to Trusts again but were unable to source consistent data on pay grades.
Why were there such differences between trusts on local spending? Any possible explanations?	We started to cover potential explanations for low local spend within the discussion section "meaning of the study and policy implications" as shown below: [p.9]<br><br>"In the case of procurement, low local spend does not necessarily imply a lack of intent. The Public services (Social Value) Act of 2012

	(enforced in 2013) requires public authorities to consider creation of economic, social and environmental value through their procurement strategies and the more recent Procurement Act of 2023 (enforced in 2025) strengthens these obligations (26, 27). However, structural constraints, including the central procurement of specialist equipment, national frameworks, and the commissioning of specialist trusts, may limit the autonomy of some organisations in shaping their supply chains (28). Some procurement also involves a national competitive bidding process, and in these instances local may not necessarily be the cheapest (12). There may also be limited availability of local suppliers and/or their capacity to participate in bidding (12, 28). However, without greater transparency and governance, it is difficult to determine whether low local spend reflects external constraints or the absence of anchor priorities within procurement strategy. An additional dimension to consider may be the distribution of local spend across providers of different capacities and whether this able to boost engagement from small and medium enterprises."<br><br>However, to elaborate further, we have added some additional details earlier in the discussion section "main findings" to explain this [p.7].<br><br>"The observed variation in local spend across trusts may be on account of inclusion of two specialist trusts which may be required to buy specialised high value equipment through procurement frameworks such as the NHS Supply chain (21), and as such have less autonomy."
I'm not quite sure what story Figure 1 is telling?	Spatial distribution of procurement spending shows regions, outside of GM ICB, where trusts might procure from. In the present case, we show that the trust with the lowest local spend procures between 10-30% from its immediate neighbour, as well as the regions in the South. We have added the

	following text to explain this in the paper [p.4].<br><br>"This spatial pattern supports visualisation of these variations, as the trust with the lowest local spend (left map) procures amounting to 10-30% of its' spend from its immediate neighbour (Lancashire and South Cumbria ICB) and from the South East. Whereas, for the trust with highest local spend (right map), its' procurement from ICBs outside of GM ICB remains under 10%."
Interested in whether there is anything geographically that might explain why Trust B is the strongest performer.	Since the trusts remain anonymised, it would not be possible to point to geographical aspects that explain performance of a specific trust.
No trusts were able to provide recent or complete data on estates and sustainability. It would be good to know what information was sought as other researchers might like to try to replicate the methods. Was there any information about why this wasn't attainable that you could include in the discussion? 'Data infrastructure and accountability' are quite superficial explanations. Have any other studies been conducted which describe large institutions estates and environmental sustainability? For example, UCL, an anchor institution in central London, uses KPIs for sustainability.<br>https://www.ucl.ac.uk/sustainable/about-us/commitments-and-strategy/ucl-sustainability-plan-2025-35 [ucl.ac.uk]	We have now added some text in the discussion section elucidating on this, which reads as follows [p.8].<br><br>"Data was requested on two other key domains of anchor activity, use of estates (measured in terms of number of bookings by community/voluntary sector) and environmental sustainability (measured as estimated annual carbon dioxide emission (tCO2) for the Trust.). However, none of the trusts were able to report data on estates, and all trusts reported data on the sustainability indicator which was part of net zero 'baseline' and hence could not be compared with the remaining analysis which was more recent."
There's a lack of citations in the para starting "In the case of procurement…"	We have now updated this with relevant citations (which were somehow omitted in the previous version). [p.9]
I would like to get a sense in the discussion of what would be the key indicators you would like if the data was available.	We have now added a paragraph in the discussion section "Strengths and weaknesses" to elucidate what additional data/indicators might have been useful. [p.8].<br><br>"As this was a retrospective study, we were limited by data we thought might be routinely collected in the NHS related to the 5 anchor pillars. If this exercise was to be done prospectively, we would want to collect nuanced data on workforce parameters such

	as, educational level, gender, access to flexible working, promotion rates, access to apprenticeships, internships, and entry-level roles, etc. A broader range of demographics and socio-economic characteristics, would allow examination of workforce representation, access to employment and training opportunities, and career progression and workplace support.”

VERSION 2 - REVIEW

Reviewer **2**

Name **Herlitz, Lauren**

Affiliation **UCL GOS Institute of Child Health, Population Policy and Practice**

Date **23-Feb-2026**

COI

The revised paper looks good, well done. Tiniest amend needed to the abstract - inconsistent use of the abbreviation of Greater Manchester (perhaps GM not needed at all?)
